# Translation and Validation of the Daily Record of Severity of Problems (DRSP) Scale in Indonesian Version

**DOI:** 10.1155/nrp/3490692

**Published:** 2025-05-15

**Authors:** Henny Dwi Susanti, Ninik Yunitri, Christina Yeni Kustanti, Min-Huey Chung

**Affiliations:** ^1^Department of Nursing, Faculty of Health Sciences, Universitas Muhammadiyah Malang, Malang, East Java, Indonesia; ^2^Faculty of Nursing, Universitas Muhammadiyah Jakarta, Jakarta, Indonesia; ^3^Study Program of Nursing Science, Sekolah Tinggi Ilmu Kesehatan, Bethesda Yakkum, Yogyakarta, Indonesia; ^4^School of Nursing, College of Nursing, Taipei Medical University, Taipei, Taiwan

**Keywords:** construct validity, premenstrual syndrome, women

## Abstract

**Objective:** This research evaluated the translation and validation of the Daily Record of Severity of Problems (DRSP) scale in the Indonesian version. This study aimed at providing a measuring tool that can be used in nursing practice, especially in detecting and managing premenstrual syndrome (PMS) in Indonesian women.

**Methods:** This study involved 315 girls in Indonesia. The DRSP used Cronbach's alpha and convenience sampling. In this study, the intraclass correlation coefficient (ICC) was utilized to assess test–retest reliability. This study used exploratory factor analysis (EFA) to evaluate factor structure and to investigate the structural model fit, and confirmatory factor analysis (CFA) was used in this study.

**Results:** The study's overall Cronbach's alpha score was 0.90. The range of the test–retest reliability ICC score was 0.72–0.90. The results of CFA for this study showed the goodness-of-fit index. The comparative fit index (CFI) was 0.90, the related fit index (RFI) was 0.81, and normed fit index (NFI) was 0.84. The value of the Kaiser–Meyer–Olkin test was 0.873, and the Bartlett's test value of sphericity was statistically significant and indicated adequate of EFA.

**Conclusion:** The result of this study showed that the DRSP scale has satisfactory reliability and validity for evaluating DRSP in Indonesia.

## 1. Introduction

Many occurrences of premenstrual syndrome (PMS) go undiagnosed and untreated because women are reluctant to seek medical attention and instead accept the symptoms as a normal part of life. In order to raise knowledge and acceptance of therapy for women's health conditions, including PMS, recent study emphasizes the significance of a culturally oriented approach [1]. Additionally, the work environment and family support are crucial in reducing the emotional strain that PMS causes. Pharmacological therapies, psychological therapy, and lifestyle modifications can all be used to detect and effectively control PMS early [2, 3]. PMS symptoms can be effectively reduced by adopting lifestyle modifications such as consistent exercise [4], a nutritious diet, and stress management [5].

PMS is characterized by various disorders, such as affective disorders, and physical and behavioral symptoms, moderate to severe. Behavioral symptoms appear during the luteal menstrual cycle and symptoms disappear within a few days after menstruation is complete [6]. Most women of reproductive age experience menstrual symptoms. Affective, physical, and behavioral symptoms that can disrupt everyday life and interfere with personal and professional activities are the hallmarks of PMS in women of reproductive age [7]. According to the epidemiology survey's findings, 80%–90% of women who menstruate have premenstrual symptoms [8]. A variety of physical, mental, and behavioral symptoms are associated with this illness; these symptoms typically manifest during the luteal phase of the menstrual cycle and go away a few days after the onset of menstruation [6]. Abdominal pains, headaches, breast soreness, mood swings, and trouble focusing are all common PMS symptoms [9]. It has a profound effect on women's quality of life, particularly about relationships with others, productivity at work, and general mental health [10].

According to the recent studies, the pathophysiology of PMS is mostly influenced by hormonal variables, particularly variations in the hormone progesterone and estrogen throughout the menstrual cycle. Mood issues associated with PMS have also been linked to neurotransmitter abnormalities, including serotonin [11, 12]. While the primary cause is biological, PMS symptoms can also be exacerbated by psychosocial factors such as stress, a lack of social support, and bad lifestyle choices [13, 14]. PMS has a major financial impact in addition to its effects on personal health. A research conducted in the United States found that PMS lowers work productivity, with absenteeism reached 45.2% [15]. Similar effects have also been observed in poorer nations, such as Indonesia, where societal stigma and restricted access to healthcare services prevent proper diagnosis and treatment.

If diagnosed correctly, PMS can be effectively managed and treated with medication and lifestyle changes. Sadly, only a small percentage of cases are identified and their symptoms managed, which may lead to lost productivity. It is possible to diagnose PMS accurately, and women of reproductive age should pay attention to two consecutive menstrual cycles since this condition enables a correlation between the onset of symptoms and the menstrual cycle [10]. The Daily Record of Severity of Problems (DRSP) is one of the valid instrument scales recommended by the Royal College of Obstetricians and Gynecologists (RCOG) guidelines [10]. When assessing PMS symptoms, tools like the DRSP have emerged as the gold standard [16].

DRSP was created to assist medical professionals in determining the degree of menstrual problems and symptoms at different phases of the menstrual cycle Endicott created DRSP in English [16], and it has subsequently been translated into German [17], Brazil [18], Chinese [19], and Japanese [20, 21] by several nations. Although the DRSP's use has grown internationally as a result of its validation and translation into other languages, Indonesian use is still quite limited. This emphasizes the necessity of creating culturally relevant tools to guarantee improved PMS diagnosis and care for Indonesian women. Social norms and culture play a significant role in how PMS is perceived and treated in Indonesia.

This study has significant relevance in the field of nursing because it provides a valid and reliable measurement tool to detect PMS symptoms in Indonesian women. In nursing practice, accurate assessment of PMS symptoms is essential to provide appropriate interventions, both in terms of education, prevention, and patient care. With the DRSP scale in the Indonesian version, nurses can more easily identify the severity of PMS and adjust the care plan according to the patient's condition.

However, women are increasingly using cognitive-behavioral therapy (CBT) to help them deal with the emotional disruptions and mood fluctuations that come with PMS [22]. Medical professionals frequently advise using hormonal contraceptives and selective serotonin reuptake inhibitors (SSRIs) for more severe symptoms. The purpose of this study is to close the gap in the literature about the reliability and validity of the DRSP's Indonesian version. By carrying out this validation, we intend to offer a trustworthy instrument for evaluating PMS symptoms in Indonesia, which will ultimately enhance the diagnosis, treatment, and standard of living for women there. The purpose of this study is to examine the validity and reliability of the Indonesian version of the instrument for evaluating premenstrual symptoms in women who are of reproductive age.

## 2. Methods

### 2.1. Design

This cross-sectional study involved girls who are still menstruating in Indonesia. This study used convenience sampling technique because this method allows efficient recruitment of participants who meet the inclusion criteria. This technique is commonly used in health research that requires voluntary participation, especially in specific populations such as girls with regular menstrual cycles and primary dysmenorrhea. Recruitment and data collection were conducted from May to December, 2023. Participants were recruited through a Google form.

### 2.2. Participants and Settings

This research design uses a cross-sectional design based on studies from Quintana and Maxwell [23]. They found that statistics produce sufficient and significant findings when the sample size is at least 200. This research uses a convenience sampling technique, and respondents must meet the inclusion criteria, including the study included participants in the 12–16 years old. In addition to agreeing and completing the informed consent form obtained from their parents, volunteers must be able to write, speak Indonesian fluently, and have had their period, primary dysmenorrhea. Girls with coronary heart disease or mental illnesses use medicine to regulate their heart rates, endometriosis, or other reproductive disorders, and participants who routinely used analgesics, oral contraceptive pills, or other hormonal medications were excluded. A total of 315 girls who fulfilled the inclusion criteria were found through recruitment.

Participants were screened during the recruitment process by asking them to confirm the first day of their menstrual cycle and asked them to record their menstrual cycle and verify their menstrual phase before participating in the study.

The sample size in this study was divided into two analyses as follows: exploratory factor analysis (EFA) and confirmatory factor analysis (CFA). The sample size for EFA was 160 participants used to explore the factor structure of the Indonesian version of the DRSP scale [24] participants, so 160 participants in this study were eligible. The sample size for CFA was 155 participants used to confirm the factor structure identified from the EFA. Previous studies have shown that a minimum of 150–200 samples are sufficient for a stable model [24]. With 155 participants, the CFA can be conducted with representative results. The sample size of 315 was determined based on a power analysis with a significance level (*α*) of 0.05 and a test power of 80%. The margin of error used was 5%.

### 2.3. Instrument

#### 2.3.1. Translation of DRSP Scale

The DRSP instrument was developed by Endicott in an English version [16]. Next, we translated the instrument into Indonesian. The translation and back-translation process in the Indonesian version of the DRSP questionnaire goes through several processes as follows: The English version of the DRSP will be translated into the Indonesian version. Next, the results of the translated version will be checked by someone who is an expert in bilingualism, both English and Indonesian. A psychologist will review the translation results to determine the DRSP questionnaire's content validity. It will be checked whether the translated questionnaire items have been used for the instrumental scale being evaluated. To verify that the translated questionnaire is identical to the original, a back-translation will be performed to view and compare it with the original. Five individuals will then be given the Indonesian version of the survey to review and assess its readability. Face validity aims to check whether the questionnaire items do not have an ambiguous impression and can be understood well.

The DRSP instrument consists of 21 items which are used to assess premenstrual symptoms both physically and emotionally. Apart from that, it is used to check functional disorders of social and life activities, where this questionnaire is used to detect the severity of premenopausal symptoms using 6 Likert scales ranging from not at all to extreme. The assessment of menstrual symptoms from these 21 symptoms will be totaled, and the score will range from a score of 21 to a score of 126.

### 2.4. Statistical Analysis

This study was examined by SPSS Version 26 and AMOS Version 18. Minimum, maximum, mean, standard deviation (SD), skewness, and kurtosis values were used to analyze the quantitative data. The degree of data asymmetry toward the mean is measured using skewness. If the skewness value is less than 3 and the kurtosis value is less than 7, then none of these metrics significantly deviates from normalcy and, consequently, from psychometric sensitivity [25].

### 2.5. Reliability

Internal consistency reliability was assessed using Cronbach's alpha, and a value higher than 0.7 indicated appropriate internal consistency [26, 27]. The intraclass correlation coefficient (ICC) is used to assess the DRSP instruments' test–retest reliability; a score of 0.75 indicates stability or sufficient test–retest reliability [28].

### 2.6. Factor Structure of DRSP

Principal axis factoring with varimax rotation was employed in EFA to determine construct validity. To assess sampling acceptability, the Kaiser–Meyer–Olkin measurement and Bartlett's test of sphericity were used to test the factor analysis [29]. To indicate capacity, the Bartlett's test value of sphericity must be significant (*p* < 0.001) and the Kaiser–Meyer–Olkin measure of sampling must be larger than 0.60 [29].

### 2.7. Construct Validity

The average variance extracted (AVE), composite reliability (CR), and factor loadings were used to assess the DRSP's convergent validity. In order to indicate adequate internal consistency, the AVE score has to be more than 0.50 [30], and the CR needed to be higher than 0.70. Furthermore, all of the factor loadings need to be greater than 0.70 in order to suggest satisfactory convergent validity.

CFA was used to evaluate the structural model fit for the DRSP. The study employed AMOS software Version 21.0 to analyze the goodness of fit. The following fit indices were computed: the goodness-of-fit index (GFI), normed fit index (NFI), comparative fit index (CFI), adjusted goodness-of-fit index (AGFI), and x2/df (the ratio of chi-square to the degree of freedom). The majority of the fit indices satisfy the criteria for SEM analysis, Doll et al. [31], Torkzadeh et al. [32], and Baumgartner and Homburg [33] also recommended that a value be acceptable if it is above 0.8, even though the values for GFI and AGFI are not above 0.9. While the RMSEA values for both models are less than 0.08, the SRMR is likewise near the threshold value [30, 34].

## 3. Result

### 3.1. Participants' Characteristics

This study included 315 total girls in Indonesia.  Table 1 shows that the mean age 13.54 with a SD of 2.46. Most girls experience menarche at the age of < 12 years, 87.3%. Most of the family income was < 2 million (89.2%), with irregular menstrual cycles at 70.5%, physical exercise at 73%, nonsmoking at 94.3%, and the majority of girls having had a history of premenstrual syndrome at 83.2%.  Table 2 shows that the value of skewness was between −0.19 and 1.05, and the value of kurtosis was from −1.09 to 0.33.

### 3.2. Psychometric Properties of DRSP

#### 3.2.1. Reliability

In the Indonesian version, the total scale's alpha coefficient for internal consistency was 0.92. According to Table 3, each subscale's Cronbach's alpha was 0.86 for Factor 1, 0.86 for Factor 2, 0.80 for Factor 3, 0.72 for Factor 4, and 0.78 for Factor 5. The ICC, which measures test–retest reliability, varied from 0.75 to 0.92 (Table 3).

#### 3.2.2. Factor Structure of DRSP

Prior to determining construct validity, this study evaluated 21 DRSP items using EFA.  Table 4 shows that the Bartlett's test value of sphericity was statistically significant (*p* < 0.001), and the Kaiser–Meier–Olkin test score was 0.873. The method of factor analysis extraction used principal component analysis (PCA) and used varimax for the rotation method. We show that the DRSP's rotating factor loadings for every item had values higher than 0.45. Six items were included in Factor 1, which was the initial factor (m5, m6, m7, m8, m9, and m10). The second factor had four items (m1, m2, m3, and m4). The third factor had five items (m17, m18, m19, m20, and m21). The fourth factor was three items (m14, m15, and m16). The fifth factor was three items (m11, m12, and m13) (Table 4).

#### 3.2.3. Construct Validity

**Figure 1:**
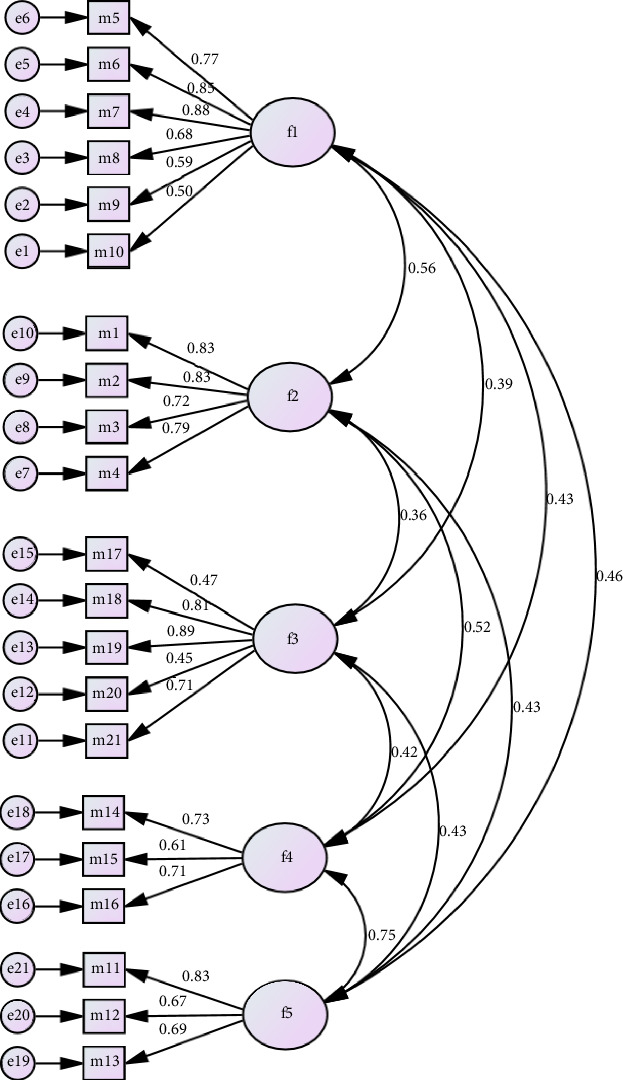
Factor structure of the DRSP. Model fit index: *X*^2^ = 534.321, df = 179, *p*=0.000, CMIN/DF = 2.985. GFI = 0.90, AGFI = 0.81, CFI = 0.90, NFI = 0.84, IFI = 0.90, TLI = 0.90, RFI = 0.81, RMSEA = 0.08.

The CR score in this study was 0.86 for Factor 1, 0.87 for Factor 2, 0.81 for Factor 3, 0.73 for Factor 4, and 0.78 for Factor 5. Favorable convergent validity was suggested by the five constructs' respective AVE values of 0.53, 0.63, 0.50, 0.50, and 0.54 (Table 3). *X*^2^ = 534.321, df = 179, *p* < 0.001, GFI = 0.90, AGFI = 0.81, CFI = 0.90, NFI = 0.84, IFI = 0.90, TLI = 0.90, RFI = 0.81, RMSEA = 0.08, and the DRSP's outcome. The model structure's goodness-of-fit findings are displayed in Figure 1.

## 4. Discussion

In this research study, the results showed that the DRSP scale in the Indonesian version produced high reliability and construct validity using the CFA. The DRSP scale produces very good reliability with a total Cronbach's alpha value of 0.90, and this value indicates a high Cronbach's alpha value. This value is almost the same as previous research conducted in several countries with Cronbach's alpha values above 0.9, such as New York with a value of 0.95 [16] and Japan with a value of 0.97 [20]. The Cronbach's alpha value for Factor 1 is 0.86, Factor 2 is 0.86, Factor 3 is 0.80, Factor 4 is 0.72, and Factor 5 is 0.78. Furthermore, the five variables in the Indonesian version of the DRSP questionnaire have high estimations of internal consistency, ranging from 0.72 to 0.86. This result is greater than 0.70, indicating that the scale has high reliability and internal consistency.

The Indonesian version of the DRSP questionnaire yields ICC findings ranging from 0.75 to 0.92, with Factor 2 having the highest value at 0.92. Factor 5 is next in line, followed by Factors 1, 3, and 4, in that order. For five components, the test–retest correlation coefficient values demonstrate good temporal stability (Table 3). A specific item's impact on the whole scale score is indicated by the item-total score correlation value. The test in this study had a high degree of reliability and was a reliable tool for measuring symptoms, as seen by the reasonably high correlation coefficient between the test–retest total DRPS scores and Cronbach's alpha values.

To extract the 21 DRSP items into 5 factors, this study used EFA. Factor 1 consists of 6 items, Factor 2 consists of 4 items, Factor 3 consists of 5 items, Factor 4 consists of 3 items, and Factor 5 consists of 3 items. However, the number of factors in this study is different from Japan, with 2 factors, where each factor consists of 11 items for Factor 1 and 10 items for Factor 2 [20].

The construct validity of the DRSP questionnaire was evaluated in this study using CFA. The Indonesian version of the DRSP questionnaire has sufficient convergent validity, according to the AVE and CR scores (Table 3). Model fit is assessed using data compatibility to determine the model structure. GFI, AGFI, CFI, NFI, IFI, TLI, RFI, and RMSEA are a few of these suitability indexes. According to the findings, DRSP displayed a model structure that was appropriate for the five variables.

This instrument has the potential to be used as a simple yet effective screening tool in identifying the severity of primary dysmenorrhea symptoms. With the validation of the Indonesian version of this instrument, health workers can use it to assess the impact of menstrual pain on patients' quality of life and design more evidence-based interventions. The use of this instrument can also contribute to further research on factors that influence menstrual pain and the effectiveness of various available treatment methods.

In addition, the application of this instrument in various healthcare settings, such as reproductive health clinics, hospitals, or community health centers, can raise awareness of the importance of menstrual pain management. With a data-driven approach from the results of this instrument's measurements, health workers can provide more appropriate recommendations regarding pharmacological and nonpharmacological therapies that are appropriate for each individual.

Furthermore, this study contributes to nursing education by providing resources that can be used in women's health assessment training. Nursing students can use the Indonesian version of the DRSP as a learning tool related to menstrual disorders and their impact on physical and psychological health. By understanding how to use this instrument, prospective nurses can be better prepared to provide evidence-based services to patients in the future.

The findings of this study can be the basis for the development of better health policies related to the management of menstrual pain, including the provision of more accessible health services for women with primary dysmenorrhea in Indonesia.

This research has limitations, including that, because this research is individual self-reporting, bias may occur. Several factors that cannot be controlled by the individual, such as the participant's emotions when answering questions, can influence the PMS symptoms reported by the participant. Another limitation is that participants reported symptoms experienced several months previously related to PMS symptoms, menarche, menstruation, and the menstrual cycle, so this could result in bias. This research has limitations, including that, because this research is individual self-reporting, bias may occur. Several factors that cannot be controlled by the individual, such as the participant's emotions when answering questions, can influence the PMS symptoms reported by the participant. Another limitation is that participants reported symptoms experienced several months previously related to PMS symptoms, menarche, menstruation, and the menstrual cycle, so this could result in bias.

## 5. Conclusions

The study's findings demonstrated that the DRSP's psychometric qualities for Indonesian women were adequate. All things considered that this study showed that the DRSP tools are highly valid and reliable for detecting premenstrual syndrome. From a nursing research perspective, the validation of the Indonesian version of the DRSP opens up opportunities for further research related to nursing interventions in PMS management. Future studies can explore the effectiveness of various nursing-based interventions, such as relaxation techniques, lifestyle changes, or psychosocial support, using the DRSP as the primary measurement tool. Thus, this study not only provides benefits for clinical nurses but also supports the development of nursing science in the aspect of women's reproductive health.

## Figures and Tables

**Table 1:** Demographic characteristics.

Characteristics	Mean (SD)	Participants for construct validity (*n* = 315)		Mean (SD)	Participants for test–retest validity (*n* = 30)	
Age	13.54 (2.46)	*N*	%	19.07 (2.39)	*n*	%
Age of menarche						
≤ 12 years		275	87.3		9	30
> 12 years		40	12.7		21	70
Family income						
< 2 million		281	89.2		9	30
≥ 2 million		34	10.8		21	70
Menstrual cycle						
Regular		93	29.5		18	60
Irregular		222	70.5		12	40
Physical exercise						
Yes		85	27.0		9	30
No		230	73.0		21	70
Smoking						
Yes		18	5.7		0	0
No		297	94.3		30	100
PMS history						
Yes		53	16.8		24	80
No		262	83.2		6	20

**Table 2:** Average score of DRSP.

Item	Min	Max	Mean	SD	Skewness	Kurtosis
m1	1	6	3.26	1.30	0.01	−0.35
m2	1	6	2.64	1.34	0.57	−0.26
m3	1	6	3.08	1.59	0.30	−1.08
m4	1	6	3.20	1.49	0.15	−0.84
m5	1	6	3.93	1.47	−0.19	−0.92
m6	1	6	3.56	1.57	0.10	−1.04
m7	1	6	3.32	1.49	0.18	−0.92
m8	1	6	2.33	1.43	1.04	0.33
m9	1	6	2.77	1.49	0.46	−0.80
m10	1	6	2.70	1.24	0.63	0.23
m11	1	6	3.44	1.42	0.15	−0.68
m12	1	6	3.21	1.58	0.16	−1.07
m13	1	6	3.63	1.45	−0.14	−0.90
m14	1	6	3.20	1.53	0.35	−0.91
m15	1	6	2.91	1.44	0.58	−0.53
m16	1	6	2.67	1.36	0.59	−0.15
m17	1	6	2.33	1.43	0.89	−0.05
m18	1	6	2.64	1.52	0.55	−0.85
m19	1	6	2.57	1.58	0.57	−0.94
m20	1	6	2.78	1.46	0.42	−0.74
m21	1	6	3.00	1.53	0.17	−1.09

**Table 3:** Reliability analysis and convergent validity.

Item	Item total correlation	Cronbach's alpha	Test–retest reliability		AVE	CR
			ICC	95% CI		
Factor 1		0.86	0.78	0.64–0.89	0.53	0.86
m5	0.70					
m6	0.73					
m7	0.78					
m8	0.65					
m9	0.61					
m10	0.50					
Factor 2		0.86	0.92	0.86–0.96	0.63	0.87
m1	0.76					
m2	0.76					
m3	0.70					
m4	0.70					
Factor 3		0.80	0.77	0.61–0.88	0.50	0.81
m17	0.44					
m18	0.67					
m19	0.72					
m20	0.47					
m21	0.65					
Factor 4		0.72	0.75	0.54–0.87	0.50	0.73
m14	0.56					
m15	0.51					
m16	0.57					
Factor 5		0.78	0.79	0.61–0.89	0.54	0.78
m11	0.63					
m12	0.58					
m13	0.63					
Total		0.90				

**Table 4:** The correlation coefficients of the DRSP items that, following varimax rotation, match the extracted factors.

Item	Factor loading				
	Factor 1	Factor 2	Factor 3	Factor 4	Factor 5
m1	0.199	**0.814**	0.144	0.165	0.093
m2	0.236	**0.823**	0.140	0.101	0.039
m3	0.133	**0.775**	0.071	0.106	0.186
m4	0.282	**0.728**	0.137	0.254	0.035
m5	**0.752**	0.254	0.074	0.013	0.231
m6	**0.821**	0.224	0.122	−0.074	0.170
m7	**0.836**	0.204	0.133	0.023	0.161
m8	**0.679**	0.181	0.144	0.253	0.063
m9	**0.657**	0.078	0.115	0.410	−0.041
m10	**0.506**	0.074	0.173	0.456	−0.008
m11	0.207	0.160	0.160	0.444	**0.623**
m12	0.136	0.113	0.115	0.168	**0.771**
m13	0.129	0.077	0.145	0.128	**0.776**
m14	0.082	0.082	0.102	**0.654**	0.443
m15	0.079	0.164	0.070	**0.669**	0.220
m16	0.099	0.291	0.174	**0.641**	0.192
m17	0.231	0.220	**0.483**	0.381	−0.215
m18	0.097	0.095	**0.823**	0.055	0.140
m19	0.149	0.047	**0.854**	0.035	0.191
m20	0.078	0.218	**0.555**	0.257	−0.041
m21	0.130	0.071	**0.768**	0.068	0.217

*Note:* Method: Factor analysis extraction: PCA, Factor analysis rotation: varimax. The bold values refer to absolute value > 0.45.

## Data Availability

Research data are not shared.
